# Setting out on a fantastic voyage to advance nanomedicine

**DOI:** 10.1038/s42003-020-0943-z

**Published:** 2020-04-30

## Abstract

We are inviting submissions of articles, perspectives, and reviews on nanomedicine, drug delivery, tumour targeting, and nanotheranostics with the aim of publishing high quality research devoted to nanotechnology for biology.

In the 1966 science fiction film “Fantastic Voyage”, a submarine and its crew are shrunk to microscopic sizes and injected into the body of an injured scientist to repair the damage to his brain and save his life. This sparked the idea in the public consciousness of a drug delivery agent which could function as an armoured vehicle, transporting its cargo to a destination (such as a tumour), moving easily through the blood stream, warding off serum proteins, dissolving blood clots on the way and ultimately killing the diseased cells. Unarguably, we have taken this idea seriously. Researchers from all over the world have tackled the enigmatic challenge of specifically targeting diseased cells and tissues heads-on. With the advent of nanotechnology, such drug delivery vehicles are now largely a reality. The implementation of nanotechnology, specifically, in the field of drug delivery, has been predicted to change the landscape of pharmaceutical and biotechnology industries, helping to develop a new armamentarium of therapeutics. With the possibility of improved delivery of poorly soluble drugs, the benefits are numerous. From active and passive targeting of specific cells or tissues to reduced off-target toxicity and real-time monitoring of in vivo therapeutic efficiency, nanomedicines hold enormous promise^1^. Although the initial optimism of quickly realising this potential has dampened a bit due to challenges with clinical translation, inefficient nanoparticle delivery, and poorly understood toxicity associated with such compounds, the effort continues to overcome these limitations and bring forth the long-awaited “wonder drug”.

We at *Communications Biology* invite and welcome such efforts to improve and transform nanomedicine. We were launched on 22 Jan 2018 to provide a home to biologists who are not defined by any one sub-area of biology^2^. This flexibility presents a specific relevance to the burgeoning field of nanomedicine, which in itself is highly interdisciplinary in nature. Material scientists, bioengineers, cell biologists, immunologists and more, all work in tandem to realise the common goal of helping the world find cures.

We at *Communications Biology* invite and welcome such efforts to improve and transform nanomedicine.

Today we publish a study by Lin et al. in which they investigate the interactions between monolayer-protected nanoparticles and model cell membranes^3^. They show four different types of nanoparticle translocation (outer wrapping, free translocation, inner attach and embedment) occurring with nanoparticle uptake. These different translocation types depend on synergism between nanoparticle size, surface charge and ligand chemistry. In another study, Prof Magzoub and his group prepared pH-responsive nanoparticles with a drug-loaded PLGA core with a cross-linked BSA corona (to avoid opsonisation)^4^. By functionalising with an ATRAM peptide that binds the cell membrane at low pH—such as tumour microenvironment—these nanoparticles display excellent in vitro and in vivo efficacy while evading recognition by macrophages. In yet another study, Li et al. describe a modified culture plate on which hiPSC-derived cardiomyocytes can form 3D self-organized tissue rings^5^. Within this ring, travelling waves of action potential were shown to spontaneously originate and promote cardiomyocytes maturation. These studies represent the diversity of topics that we are interested in, ranging from nanoparticle-membrane interaction and stimuli responsive drug carriers to tissue engineering.

Indeed, finding cures has taken an altogether different meaning today as the world currently stands, grappling under the grim reality of a global pandemic. COVID-19 cases are on the rise and life, as we knew it, has changed dramatically within a matter of weeks. Nearly everyone is affected in some way by the spreading novel coronavirus and multiple countries have gone into complete lockdown. Implementing social isolation is hard, and we appreciate that scientists are enduring the additional challenges of working from home, laboratories being closed and facing imminent funding issues. But cliché as it may seem, the key is to not lose hope. We encourage you to dwell on those incipient research ideas, read extensively for inspiration and brush up the manuscripts that need finalising. And as we at *Communications Biology* embark on our own fantastic voyage, we would like to think of you as fellow travellers. Who knows if one of those research ideas may be the key to overcoming the current pandemic or other challenges to come.
